# Integrated transcriptome and co-expression network analysis revealed the molecular mechanism of cold tolerance in japonica rice at booting stage

**DOI:** 10.3389/fpls.2025.1629202

**Published:** 2025-07-03

**Authors:** Jiaqi Wang, Ning Chen, Jiaying Li, Chengxin Li, Guanzheng Fu, Fuying Liu, Hongqiang Zhao, Yingying Liu, Weihan Jiang, Tianyu Xia, Jie Chen, Hualong Liu, Hongliang Zheng, Luomiao Yang, Detang Zou, Jingguo Wang, Wei Xin

**Affiliations:** ^1^ Key Laboratory of Germplasm Enhancement and Physiology & Ecology of Food Crop in Cold Region, Ministry of Education/College of Agriculture, Northeast Agricultural University, Harbin, China; ^2^ Harbin Academy of Agricultural Sciences, Harbin, China; ^3^ Harbin No.9 High School, Harbin, China; ^4^ Tianjin Tianlong Science & Technology Company Limited, Tianjin, China

**Keywords:** rice, panicle formation, cold tolerance, transcriptome, WGCNA

## Abstract

**Background:**

Cold stress during the booting stage severely reduces rice yield by impairing pollen development and seed-setting rates. To uncover the molecular basis of cold tolerance, we analyzed phenotypic and transcriptomic data from 14 japonica rice varieties under cold stress, combined with WGCNA.

**Results:**

The results demonstrated that cold stress significantly decreased yield traits, particularly seed-setting rate—a reliable cold tolerance indicator. Transcriptome analysis identified 6,240 and 7,996 DEGs in cold-tolerant and cold-sensitive varieties, respectively, with 1,875 core genes enriched in key pathways like plant hormone signaling and MAPK cascades.

**Conclusion:**

WGCNA analysis identified a seed-setting rate-associated blue module, which contained 10 highly connected candidate genes and 20 core transcription factors potentially involved in cold tolerance. This study provides novel insights into the molecular mechanisms of cold tolerance in rice and offers valuable targets for molecular breeding of cold-resistant cultivars.

## 1 Introduction

Rice (*Oryza sativa L.*) is one of the world’s most critical staple food crops and a primary dietary component for over half of the global population. As a key pillar of worldwide food security, rice requires enhanced yield and quality to address global climate change and population growth. Originating in tropical regions, it exhibits greater sensitivity to low temperatures than other crops (Wang et al., 2003; Zhang et al., 2014; Wang et al., 2016). This vulnerability is particularly pronounced during the booting stage, where exposure to chilling injury can cause irreversible yield losses (Li et al., 2025; Fu et al., 2025). Chilling injury during the booting stage disrupts another development, with the anther being one of the most cold-sensitive organs in rice. The pollen mother cells are highly sensitive to temperature fluctuations during the transition from the tetrad stage to the early uninucleate stage, a critical window that coincides with tapetum development. Cold stress interferes with programmed tapetal degradation, leading to abnormal enlargement of tapetal cells and consequently disrupting nutrient supply to developing pollen mother cells. This impairment disrupts carbohydrate metabolism, leading to pollen sterility through incomplete maturation and dispersal failure. These physiological alterations are evidenced by elevated spikelet sterility, decreased seed-setting rate, and ultimately reduced grain yield and quality (Sharma and Nayyar, 2016; Han et al, 2024). Research indicates that in temperate regions, chilling injury can reduce rice yields by 30-40% (Andaya et al., 2003). In major Chinese rice cultivation zones such as the Middle-lower Yangtze River basin and northeastern regions, seasonal chilling injury causes annual production losses of 3–5 million metric tons (Zhu et al., 2015). Therefore, developing cold-tolerant rice germplasm represents a critical strategy to mitigate yield losses caused by chilling injury, which will not only facilitate the elucidation of molecular mechanisms underlying cold stress responses in rice but also accelerate the breeding of novel cold-tolerant cultivars.

Chilling injury represents an extremely complex physiological phenomenon, while the mechanisms underlying cold stress resistance constitute a multilayered and sophisticated response system involving coordinated actions across physiological metabolism, hormonal signaling, and molecular pathways. At the physiological level, plants enhance cold stress adaptation through increased activity of reactive oxygen species (ROS)-scavenging enzymes such as superoxide dismutase (SOD), peroxidase (POD), and catalase (CAT), along with accumulation of compatible solutes including soluble sugars, proline, and functional proteins (Ding et al., 2019). At the molecular level, cold stress can activate certain Ca2^+^ -permeable channels and Ca2^+^ transporters to initiate abiotic stress responses. The mutation of *OsCNGC9, OsCNGC14* or *OsCNGC916* in rice will lead to the increase of hydrogen peroxide accumulation and cell death under cold stress, while *OsCNGC9* can be phosphorylated and activated by *OsSAPK8* to enhance the sensitivity to Ca2^+^ (Wang et al., 2021). Furthermore, heterotrimeric G-protein signaling participates in cold adaptation through *COLD1* interaction with the Gα subunit (*RGA1*), which activates calcium channels to promote Ca²&^+^ influx and consequently improves cold-tolerance (Zhu, 2016). Hormones can not only directly regulate physiological metabolic processes, but also act as signaling molecules to regulate the expression of cold tolerance-related genes by activating low temperature response pathways (Ma et al., 2025). Under cold stress, activated ABA biosynthetic genes elevate endogenous ABA levels to initiate defense mechanisms (Li et al., 2021). Subsequent stress relief restores ABA catabolism, maintaining homeostasis essential for survival and normal development (Li et al., 2025). Similarly, gibberellins (GAs) positively regulate cold tolerance during the rice booting stage, as evidenced by the cold-sensitive phenotypes of GA biosynthesis mutants (*sd1*, *d35*) and signaling mutants (*gid1*, *slr1*) (Sakata et al., 2014 ). *CTB5* modulates anther GA homeostasis by targeting *OsGA2ox6* and *OsGA3ox1*, with exogenous GA application rescuing seed setting rate in *CTB5* knockout/overexpression lines under cold stress (Guo et al., 2025). The conserved MAPK cascade (MAPKKK→MAPKK→MAPK) mediates cold signal transduction through sequential phosphorylation events. In Arabidopsis, *MAPKK4/5-MAPK3/6* negatively regulates cold tolerance by phosphorylating *ICE1* (Petersen et al., 2000), while *OsMAPK6* positively regulates cold tolerance in rice seedlings (Liu et al., 2024)

Transcriptome sequencing (RNA-seq) has been widely used in gene mining (Barah et al., 2013; Li et al., 2018; Kang et al., 2020; Sun et al., 2020) to comprehensively analyze gene expression profiles, while weighted gene co-expression network analysis (WGCNA) can construct gene regulatory networks based on expression data and mine functional modules and key genes. The joint analysis of the two has become a powerful tool for revealing complex biological processes (Luo et al., 2022; Li et al., 2023; Lin et al., 2025). Previous studies showed that high temperature could affect the grain filling during grain filling, and revealed the possible metabolic pathways that caused the shortage of storage substances by transcriptome analysis (Yamakawa and Hakata, 2010). Zhou’s team applied WGCNA to construct a co-expression network of differentially expressed genes in Beckmannia syzigachne under drought and rewatering conditions, identifying key drought-tolerance genes (Zhou et al., 2021). Another study revealed the mechanism of Cd accumulation mediated by pectin demethylation modification in lettuce roots using transcriptome combined with WGCNA analysis (Zhang et al., 2025).

Although the mapping of rice cold tolerance genes at booting stage has been reported in succession, so far, only a few cold tolerance genes at booting stage have been isolated, such as *CTB1* (Saito et al., 2010), *CTB2* (Li et al., 2021), *CTB3* (Li et al., 2025), *CTB4a* (Zhang et al., 2017), *APXa* (Sato et al., 2011), *MAPK3* (Lou et al., 2022), *LEA9* (Liu et al., 2018), *bZIP73* (Liu et al., 2018), *LTT1* (Xu et al., 2020), *WRKY53* (Tang et al., 2022), *MKKK70* (Mei et al., 2022), *qCTB7* (Yang et al., 2023) and *COG3* (Liu et al., 2024). *CTB1* was the first cloned gene specifically conferring cold tolerance during the booting stage in rice (Saito et al., 2010). Therefore, it is urgent to systematically explore more potential cold-tolerant gene resources through the joint analysis of transcriptomics and WGCNA. This integration strategy can identify key functional modules and core regulatory factors (Hub genes) through a co-expression network, and provide a more comprehensive candidate gene pool for the study of the molecular mechanism of rice cold tolerance.

## 2 Materials and methods

### 2.1 Plant materials

In 2022, the cold tolerance of the natural population composed of 278 japonica rice germplasm resources was identified in the artificial climate chamber at the booting stage. According to the relative seed setting rate under the treatment and control conditions, 14 japonica rice varieties with continuous variation of cold tolerance were selected as the experimental materials of this study (Additional file 1: **Supplementary Table S1**).

### 2.2 Determination of yield and its components

14 japonica rice seeds were soaked and germinated on April 7, sowed and cultivated in greenhouse on April 15, and transplanted into pots on May 20. During the booting stage of japonica rice, the japonica rice was transferred to the artificial climate chamber for 17(± 0.5) °C, continuous 5 days of cold air stress treatment, and the photoperiod was the same as that of the outside world. The japonica rice under normal conditions at booting stage was used as the control, and the phenotype was determined after maturity. Five single plants of japonica rice under normal conditions and five single plants of japonica rice under low temperature treatment were selected to investigate their yield per plant, effective panicle number per plant, total grain number per panicle, seed setting rate and 1000-grain weight. The significance analysis of each trait of each japonica rice variety was carried out.

### 2.3 Transcriptome sequencing

When 14 japonica rice varieties were subjected to low-temperature treatment at the booting stage for 0 d and 3 d, young panicles were collected at different time points for transcriptome analysis. The harvested samples were wrapped in sterilized tin foil, flash-frozen in liquid nitrogen, and then stored at −80°C. Total RNA was extracted using TRarsZolUpRNA Kit (Beijing, China). Firstly, the quality control of 14 samples was measured by OneDrop 1000 + instrument. The RNA content and A260/A280 were measured by OneDrop 1000 + instrument. Three complete target bands were detected by agarose gel electrophoresis. Secondly, the samples were sent to the sequencing company for testing, and the qualified samples were used to construct the library. The clean reads at different temperatures were compared with the control genome by HISAT2 technology (Kim et al., 2019), and the number of genes was obtained by FeatureCounts analysis. Finally, the FPKM values of each gene were calculated by gene length, read counts and other methods to reflect the expression level of the gene.

### 2.4 Detection and analysis of differentially expressed genes

To identify differentially expressed genes (DEGs) between cold-tolerant and cold-sensitive varieties under cold stress, read count data were analyzed in R using DESeq2. The P-values were adjusted for multiple hypothesis testing using the false discovery rate (FDR). DEGs were screened based on the following thresholds: |log&_2_FC| > 1 (indicating a twofold change in expression); FDR < 0.05 (for statistical significance). To characterize the transcriptional changes, Gene Ontology enrichment analysis and KEGG pathway analysis were performed. Functional enrichment of DEGs was conducted using the Maiwei online platform, with a significance threshold of FDR < 0.05. For KEGG enrichment analysis, KOBAS (v3.0) was used, applying the same threshold (FDR < 0.05)

### 2.5 Screening of cold-tolerant gene sets

Genes that are specifically expressed in cold-tolerant varieties, as well as those expressed in both cold-tolerant and cold-sensitive varieties but exhibiting significantly higher differential expression levels in cold-tolerant varieties [FC (cold-tolerant/cold-sensitive) ≥ 1.5], were selected as the cold-tolerant gene set.

### 2.6 Construction of co-expression networks and gene mining

First, low-expression genes were filtered during the construction of the co-expression network to ensure the accuracy and efficiency of the analysis. Subsequently, pairwise correlation analysis was performed between genes to establish a gene co-expression similarity matrix. An appropriate soft threshold power (β) was selected to ensure the gene expression relationships conformed to a scale-free network. The β value was calculated using the pick Soft Threshold function from the WGCNA package (Yamakawa and Hakata, 2010) The selection criteria were as follows: β was tested within the range of 1–20, and the optimal β was chosen based on achieving a sufficient level of gene connectivity while maintaining the scale-free topology fit index (R²) close to 0.9. With R² = 0.9, a weighting coefficient (β) of 16 was selected for network construction, as this value ensures biologically meaningful gene correlations (Yamakawa and Hakata, 2010).

### 2.7 Data analysis

Phenotypic data obtained in this study were organized using Microsoft Excel and subjected to descriptive statistical analysis. Correlation analysis was performed using IBM SPSS Statistics 26.0 (SPSS Inc., Chicago, IL, USA). Transcriptomic data were analyzed and visualized using the Metware Online Platform (https://cloud.metware.cn/), while co-expression networks of transcription factors and candidate genes were constructed and visualized using the Cytoscape software package.

## 3 Results

### 3.1 Impact of cold stress during the booting stage on yield formation in japonica rice

**Figure 1:**
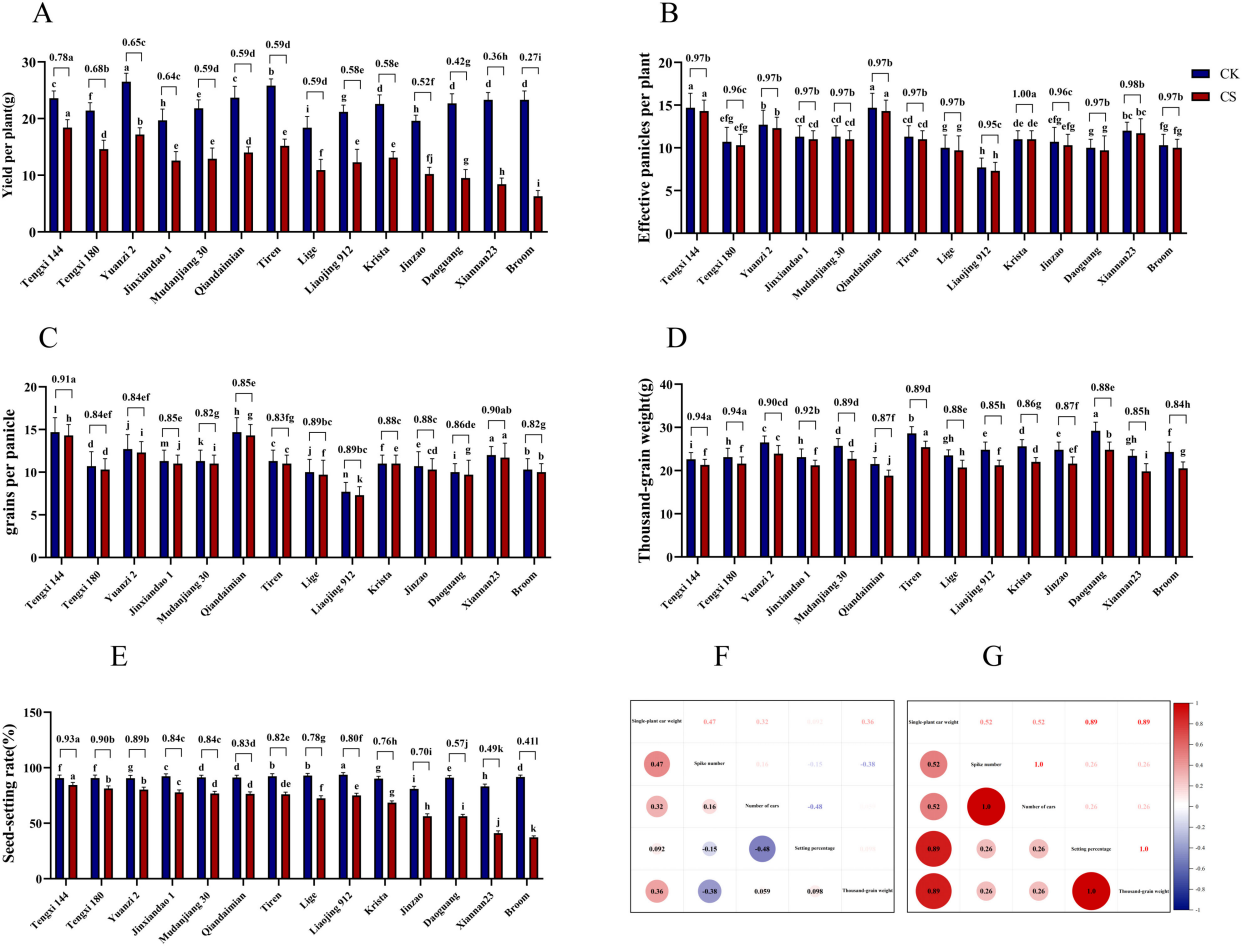
Yield and its components of 14 japonica rice varieties under different treatments and their correlation analysis. **(A-E)** Yield and its components of 14 japonica rice varieties, In **(A-E)** figures, The lowercase in the figure are important test labels obtained by one-way ANOVA.different lowercase letters in the figure indicate significant differences (*P* < 0.05) in yield components among treatments, while shared letters denote no significant difference; the value on the adjacent column of CK and CS is the ratio of CS to CK(CS/CK). **(F)** Correlation analysis of yield and its components in japonica rice under normal conditions, **(G)** Correlation analysis of yield and its components in japonica rice after cold treatment.

This study investigated the yield and its components of japonica rice varieties under different treatments during the booting stage, with normal temperature as the control group and low temperature as the treatment group. We analyzed significant differences in various yield-related indicators. The results showed that the yield per plant of 14 rice varieties under control conditions ranged from 18.4 to 26.5 g, whereas low-temperature treatment significantly reduced yields to 6.3–18.4 g, with relative yield per plant ranging from 0.27 to 0.78. The study demonstrated that low-temperature treatment during the booting stage significantly decreased all yield components, including yield per plant, Spike number, grains per panicle, seed-setting rate, and 1000-grain weight in all japonica rice varieties. Among these, Tengxi 144 showed superior performance in relative yield per plant, relative grains per panicle, and relative seed-setting rate, exhibiting significant differences compared to other varieties. In contrast, Broom exhibited the lowest relative seed-setting rate, significantly differing from other varieties. These findings collectively indicate that the seed-setting rate demonstrated the most significant changes following low-temperature treatment, while other phenotypic indicators showed relatively minor alterations (**Figures 1A–E**).

Further correlation analysis of yield and its components revealed that under normal conditions, yield per plant of japonica rice showed significant positive correlations with effective panicle number and grains per panicle. However, after low-temperature treatment, yield exhibited significant positive correlations with effective panicle number and seed-setting rate (**Figures 1F, G**), while relative seed-setting rate also showed a significant positive correlation with relative yield (Additional file 7: **Supplementary Figure S1**). This suggests that low-temperature treatment significantly affects seed-setting rate, which emerges as a crucial factor influencing japonica rice yield under cold stress during the booting stage, and can serve as a primary indicator for evaluating cold tolerance. Based on relative seed-setting rate, Tengxi 144, Tengxi 180, and Yuanzi 2 were identified as cold-tolerant varieties, while Daoguang, Xiannan 23, and Broom were classified as cold-sensitive varieties.

### 3.2 Transcriptome analysis of cold-tolerant and cold-sensitive rice varieties under booting-stage cold stress

**Figure 2:**
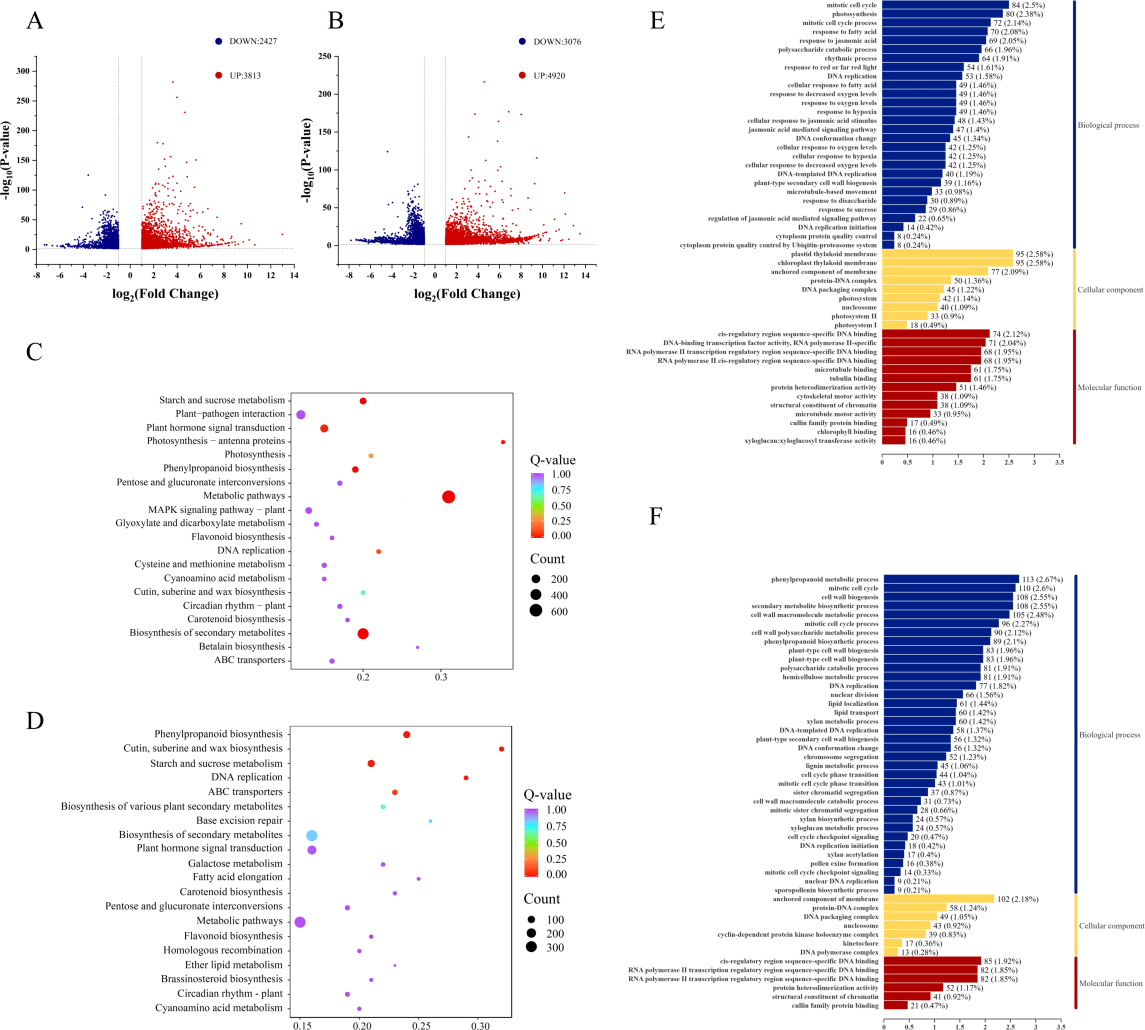
Analysis of DEGs between cold-tolerant and cold-sensitive varieties. **(A)** Volcano plot of DEGs in cold-tolerant varieties, red represents significantly upregulated genes (FDR <0.05 and log&_2_FC> 1), blue represents significantly downregulated genes (FDR <0.05 and |log&_2_FC| <-1), and gray represents non-significant genes, **(B)** Volcano plot of DEGs in cold-sensitive varieties, The color label is the same as **(A, C)** KEGG pathway enrichment of DEGs in cold-tolerant varieties, the vertical axis is the name of the pathway, and the horizontal axis is the enrichment significance. The color represents the category of the pathway, **(D)** KEGG pathway enrichment of DEGs in cold-sensitive varieties, its label is the same as **(C, E)** GO functional enrichment of DEGs in cold-tolerant varieties, the horizontal axis is the number of enriched genes, and the color represents GO categories, **(F)** GO functional enrichment of DEGs in cold-sensitive varieties, its label is the same as **(E)**.

Transcriptome analysis of 14 japonica rice cultivars revealed distinct molecular responses to cold stress between cold-tolerant and cold-sensitive varieties. Under cold stress conditions, cold-tolerant cultivars exhibited 6,240 differentially expressed genes (DEGs) (3,813 upregulated and 2,427 downregulated), while cold-sensitive cultivars showed a more pronounced response with 7,996 DEGs (4,920 upregulated and 3,076 downregulated) (**Figures 2A, B**).

Functional enrichment analysis of DEGs in both cold-tolerant and cold-sensitive varieties was performed through Gene Ontology (GO) and KEGG pathway analyses. In cold-tolerant varieties, GO analysis revealed significant enrichment of DEGs in Biological Processes (BP) including mitotic cell cycle, photosynthesis, and mitotic cell cycle processes. For Cellular Components (CC), DEGs were enriched in thylakoid membranes (chloroplast), thylakoid membranes (plastid), and membrane-anchored components. Regarding Molecular Function (MF), significant enrichment was observed for sequence-specific DNA binding in cis-regulatory regions and DNA-binding transcription factor activity (RNA polymerase II-specific) (**Figure 2E**).

In contrast, DEGs from cold-sensitive varieties showed significant enrichment in BP terms including phenylpropanoid metabolic processes, mitotic cell cycle, and secondary metabolite biosynthetic processes. For CC, enrichment was observed in membrane-anchored components, protein-DNA complexes, and DNA packaging complexes. In MF, DEGs were enriched for sequence-specific DNA binding in cis-regulatory regions and sequence-specific DNA binding in RNA polymerase II transcription regulatory regions (**Figure 2F**).

KEGG pathway analysis further revealed that DEGs in cold-tolerant varieties were significantly enriched in pathways including starch and sucrose metabolism, plant hormone signal transduction, photosynthesis-antenna proteins, photosynthesis, metabolic pathways, MAPK signaling pathway-plant, and glycerophospholipid metabolism (**Figure 2C**). In contrast, DEGs from cold-sensitive varieties showed significant enrichment in pathways such as phenylpropanoid biosynthesis, and cutin/suberin/wax biosynthesis (**Figure 2D**).

**Figure 3:**
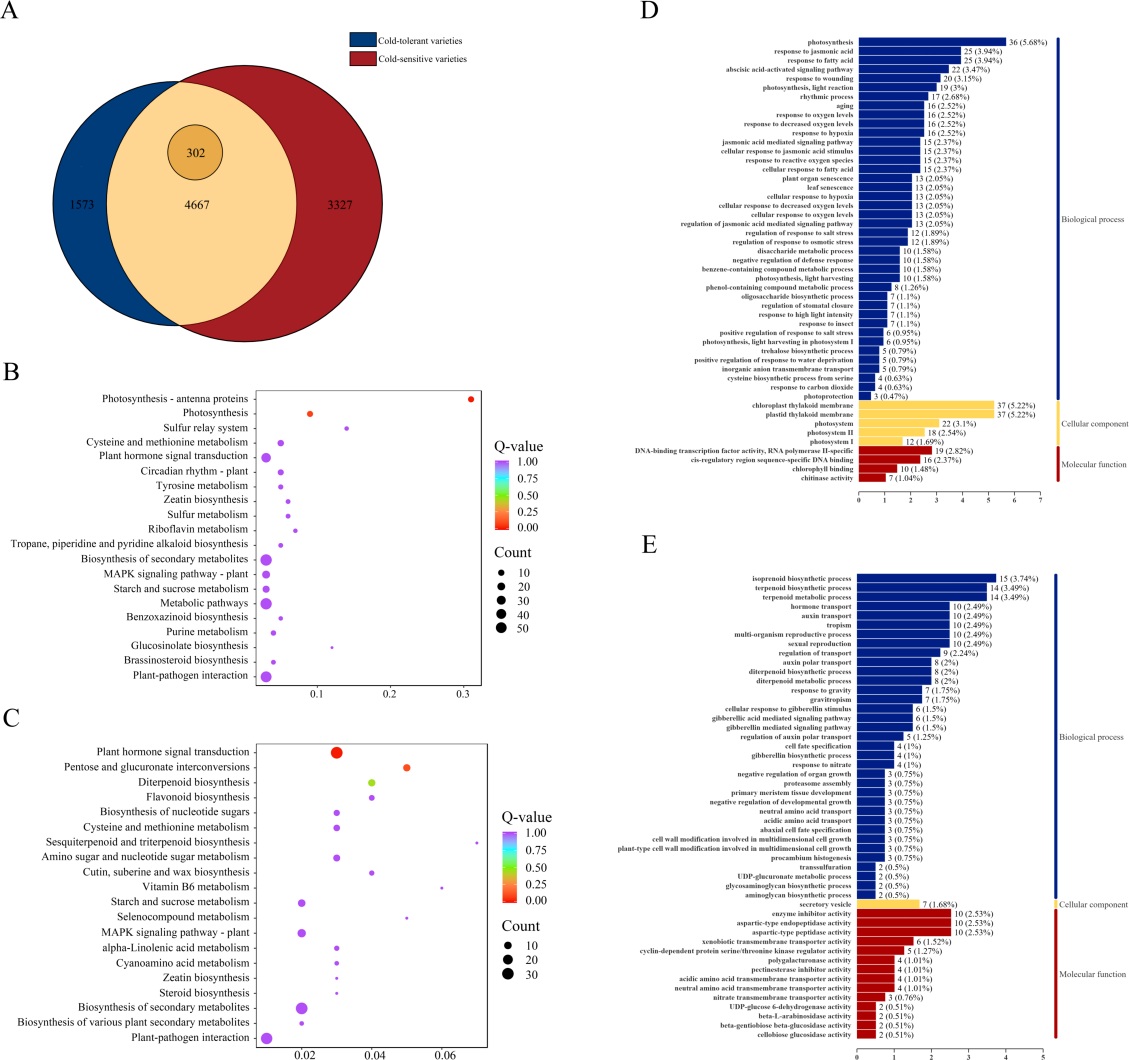
Screening and functional characterization of cold-tolerance-related gene sets. **(A)** Statistics of DEGs between cold-tolerant and cold-sensitive varieties under cold stress, **(B)** GO functional enrichment analysis of up-regulated DEGs in the cold-tolerance-related gene set, its labeling is the same as **Figure 2C**. **(C)** GO functional enrichment analysis of down-regulated DEGs in the cold-tolerance-related gene set, its label is the same as **(B, D)** KEGG pathway enrichment analysis of up-regulated DEGs in the cold-tolerance-related gene set, its labeling is the same as **Figure 2E**. **(E)** KEGG pathway enrichment analysis of down-regulated DEGs in the cold-tolerance-related gene set.

To identify core genes directly associated with cold tolerance, we applied the following stringent selection criteria: (1) genes specifically expressed only in cold-tolerant varieties, and (2) genes expressed in both cold-tolerant and cold-sensitive varieties but showing significantly higher differential expression levels in cold-tolerant varieties [FC (cold-tolerant/cold-sensitive) ≥ 1.5]. This screening identified 1,573 genes that were specifically and differentially expressed only in cold-tolerant varieties, along with 302 genes that were expressed in both varieties but exhibited significantly higher expression levels in cold-tolerant varieties. Ultimately, we obtained a core cold-tolerance gene set comprising 1,875 genes (**Figure 3A**).

To functionally characterize these 1,875 core cold-tolerance genes, we conducted separate GO and KEGG enrichment analyses for both upregulated and downregulated DEGs. The GO analysis of upregulated DEGs revealed significant enrichment in key MF including DNA-binding transcription factor activity (RNA polymerase II-specific) and chlorophyll binding, with CC predominantly localized to plastid and chloroplast thylakoid membranes. These genes were primarily involved in critical BP such as photosynthesis and responses to jasmonic acid and fatty acids (**Figure 3D**). Among the down-regulated DEGs, significant enrichments were found in MF such as enzyme inhibitor activity, aspartic-type endopeptidase activity, and aspartic-type peptidase activity. For CC, the secretory vesicles were significantly enriched. In BP, enrichments included isoprenoid biosynthetic process, terpenoid biosynthetic process, and terpenoid metabolic process (**Figure 3E**). These findings collectively emphasize the pivotal roles of plant hormone signaling, MAPK pathways, photosynthetic processes, and specific metabolic networks (particularly starch and sucrose metabolism) in establishing cold tolerance in japonica rice.

KEGG pathway enrichment analysis demonstrated that the upregulated DEGs were significantly enriched in starch and sucrose metabolism, plant hormone signal transduction, photosynthesis-antenna proteins, photosynthesis, plant-pathogen interaction, MAPK signaling pathway-plant, carbon metabolism, diterpenoid biosynthesis, cysteine and methionine metabolism, phenylpropanoid biosynthesis, amino acid biosynthesis, cofactor biosynthesis, and auxin-activated signaling pathway (**Figure 3B**). In contrast, the downregulated DEGs showed significant enrichment in plant hormone signal transduction, biosynthesis of secondary metabolites, plant-pathogen interaction, metabolic pathways, MAPK signaling pathway-plant, starch and sucrose metabolism, pentose and glucuronate interconversions, diterpenoid biosynthesis, amino sugar and nucleotide sugar metabolism, cysteine and methionine metabolism, cofactor biosynthesis, and nucleotide sugar biosynthesis (**Figure 3C**).

### 3.3 Response of plant hormone signal transduction metabolic pathway to cold stress

**Figure 4:**
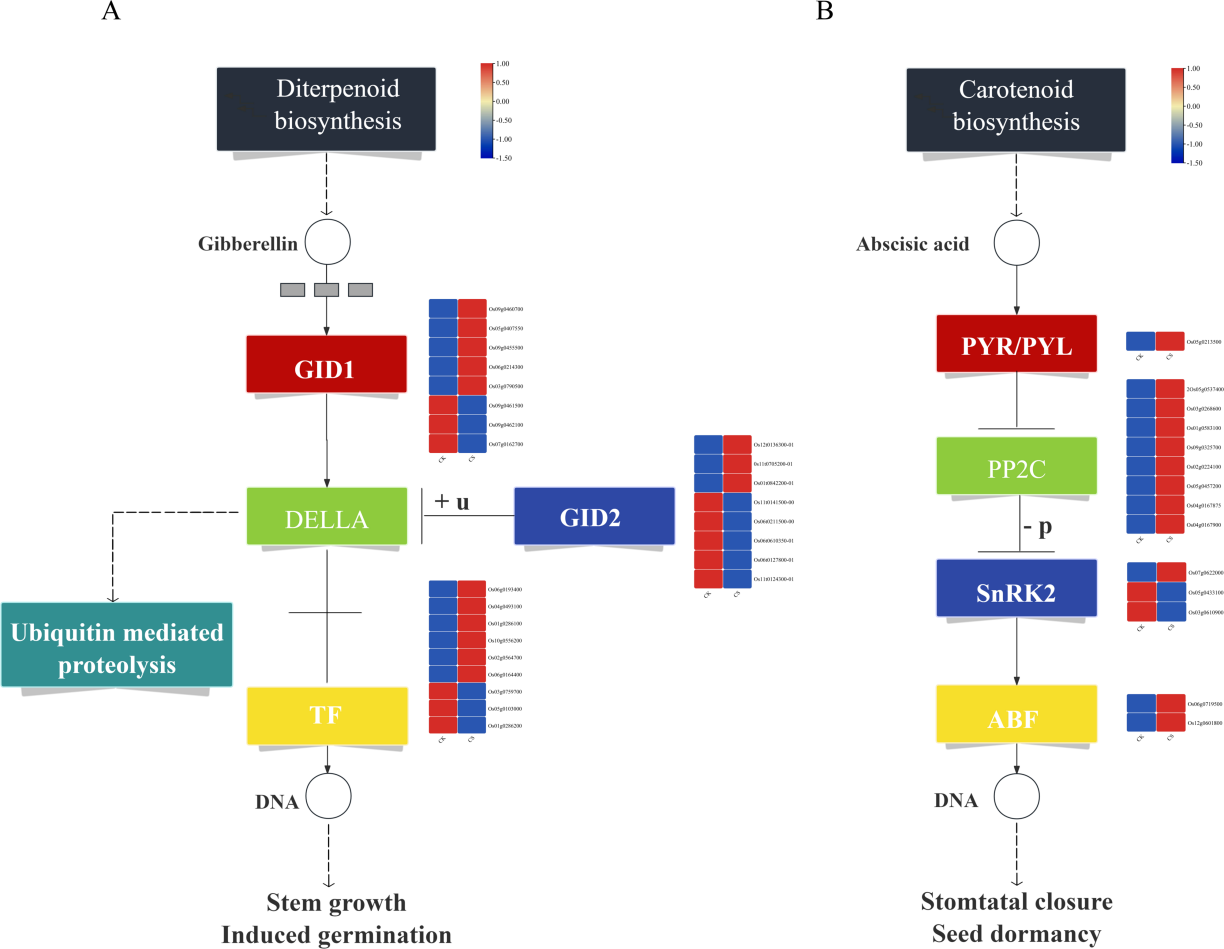
Plant hormone signal transduction and metabolic pathways. **(A)** GA metabolic pathway, **(B)** ABA metabolic pathway.

KEGG functional enrichment analysis revealed significant enrichments in metabolic pathways closely associated with cold tolerance, including plant hormone signal transduction and MAPK signaling pathway - plant. Under cold stress, a total of 25 DEGs related to gibberellin (GA) metabolism were identified (**Figure 4A**). Among these, 8 genes encode GID1 receptor proteins, with 5 genes (*Os09g0460700*, *Os05g0407550*, *Os09g0455500*, *Os06g0214300*, *Os03g0790500*) showing significantly up-regulated expression and 3 genes (*Os09g0461500*, *Os09g0462100*, *Os07g0162700*) exhibiting significantly down-regulated expression. Additionally, 8 genes encode DELLA proteins, which are transcriptional repressors in the GA signaling pathway, with 3 genes (*Os12g0136300*, *Os11g0705200*, *Os01g0842200*) significantly up-regulated and 5 genes (*Os11g0141500*, *Os06g0211500*, *Os06g0610350*, *Os06g0127800*, *Os11g0124300*) significantly down-regulated. Furthermore, 9 transcription factors associated with GA metabolism were identified, including 8 bHLH family members (*Os06g0193400*, *Os04g0493100*, *Os01g0286100*, *Os10g0556200*, *Os02g0564700*, *Os06g0164400*, *Os03g0759700*, *Os05g0103000*) and 1 hypothetical protein (*Os01g0286200*). Among these, 6 transcription factors were up-regulated, while 3 were down-regulated (Additional file 2: **Supplementary Table S2**).

Under cold stress, 14 DEGs related to abscisic acid (ABA) metabolism were identified (**Figure 4B**). Among these, 1 gene encodes the PYR/PYL receptor protein (*Os05g0213500*), which was significantly up-regulated. Additionally, 8 genes encode negative regulators of the ABA pathway, specifically PP2C proteins (*Os04g0167900*, *Os05g0537400*, *Os03g0268600*, *Os01g0583100*, *Os09g0325700*, *Os02g0224100*, *Os05g0457200*, *Os04g0167875*), all of which were up-regulated. Furthermore, 3 genes encode positive regulators of the ABA pathway, namely SnRK2 proteins: *Os07g0622000* (up-regulated) and *Os05g0433100*, *Os03g0610900* (down-regulated). Notably, *Os03g0610900* is a known cold-tolerance gene involved in cold stress regulation. Additionally, 2 transcription factors associated with the ABA pathway were identified, both belonging to the bZIP family (*OsbZIP54*, *Os12g0601800*). Among these, *OsbZIP54* is a known cold-tolerance gene, and both transcription factors were up-regulated (Additional file 3: **Supplementary Table S3**).

### 3.4 Response of MAPK signaling pathway to cold stress

**Figure 5:**
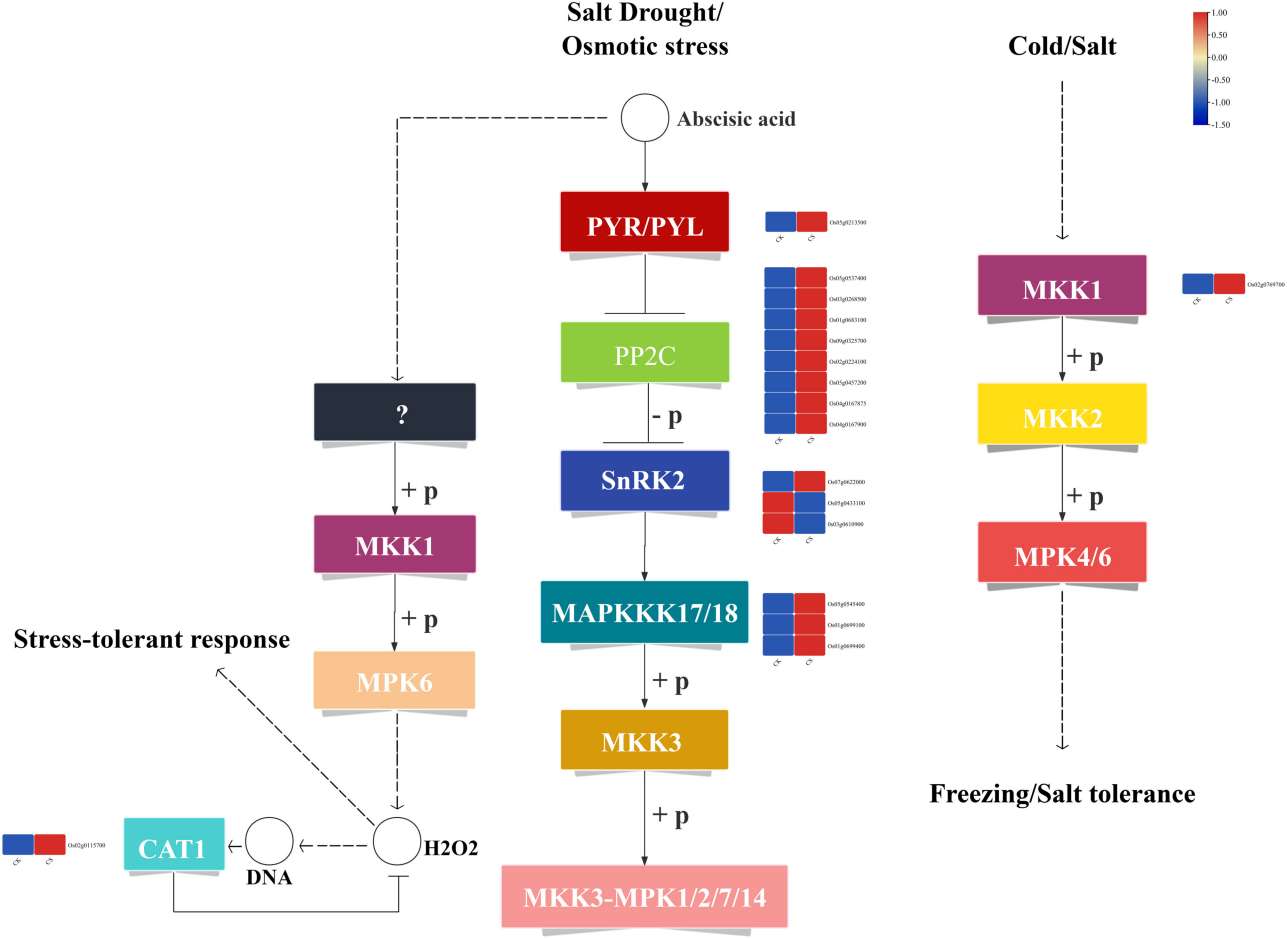
MAPK signaling pathway.

A total of 17 DEGs associated with the MAPK signaling pathway were identified under cold stress (**Figure 5**). Among these, 1 gene encodes the PYR/PYL receptor protein (*Os05g0213500*), which was significantly up-regulated. Additionally, 8 genes encode negative regulators of the MAPK pathway, specifically PP2C proteins (*Os04g0167900*, *Os05g0537400*, *Os03g0268600*, *Os01g0583100*, *Os09g0325700*, *Os02g0224100*, *Os05g0457200*, *Os04g0167875*), all of which were significantly up-regulated. Furthermore, 3 genes encode positive regulators of the MAPK pathway, namely SnRK2 proteins, with 1 gene (*Os07g0622000*) significantly up-regulated and 2 genes (*Os05g0433100*, *Os03g0610900*) significantly down-regulated. Notably, *Os03g0610900* is a known cold-tolerance gene involved in cold stress regulation. In addition, 3 genes encode the MAPKKK17/18 signal protein kinases (*Os05g0545400*, *Os01g0699100*, *Os01g0699400*), all of which were significantly up-regulated. Among these, *Os01g0699100* is a known cold-tolerance gene. Moreover, 1 gene encoding MKK1 (*Os02g0769700*) was significantly up-regulated. In the cold stress response pathway, 1 gene encoding *CAT1* (*Os02g0115700*) was significantly up-regulated, with *Os02g0115700* being a known cold-tolerance gene (Additional file 4: **Supplementary Table S4**).

### 3.5 Screening of candidate modules and construction of regulatory networks

To investigate the cold tolerance mechanisms in japonica rice, we employed weighted gene co-expression network analysis (WGCNA) using transcriptome data from 14 japonica varieties subjected to cold treatment at the booting stage. A total of 20,093 genes were incorporated into network construction, with the soft thresholding power (β) determined as 16 based on scale-free topology criterion (R² = 0.9) (Additional file 8: **Figure 2**). Following exclusion of the poorly clustered Gray module, 18 distinct co-expression modules were ultimately identified. Hierarchical clustering revealed two primary module clusters, with correlation heatmap visualization demonstrating negative associations (blue gradient, 0-0.5) and positive associations (red gradient, 0.5-1), where intensity correlates with association strength (Additional file 9: **Supplementary Figure S3**).

**Figure 6:**
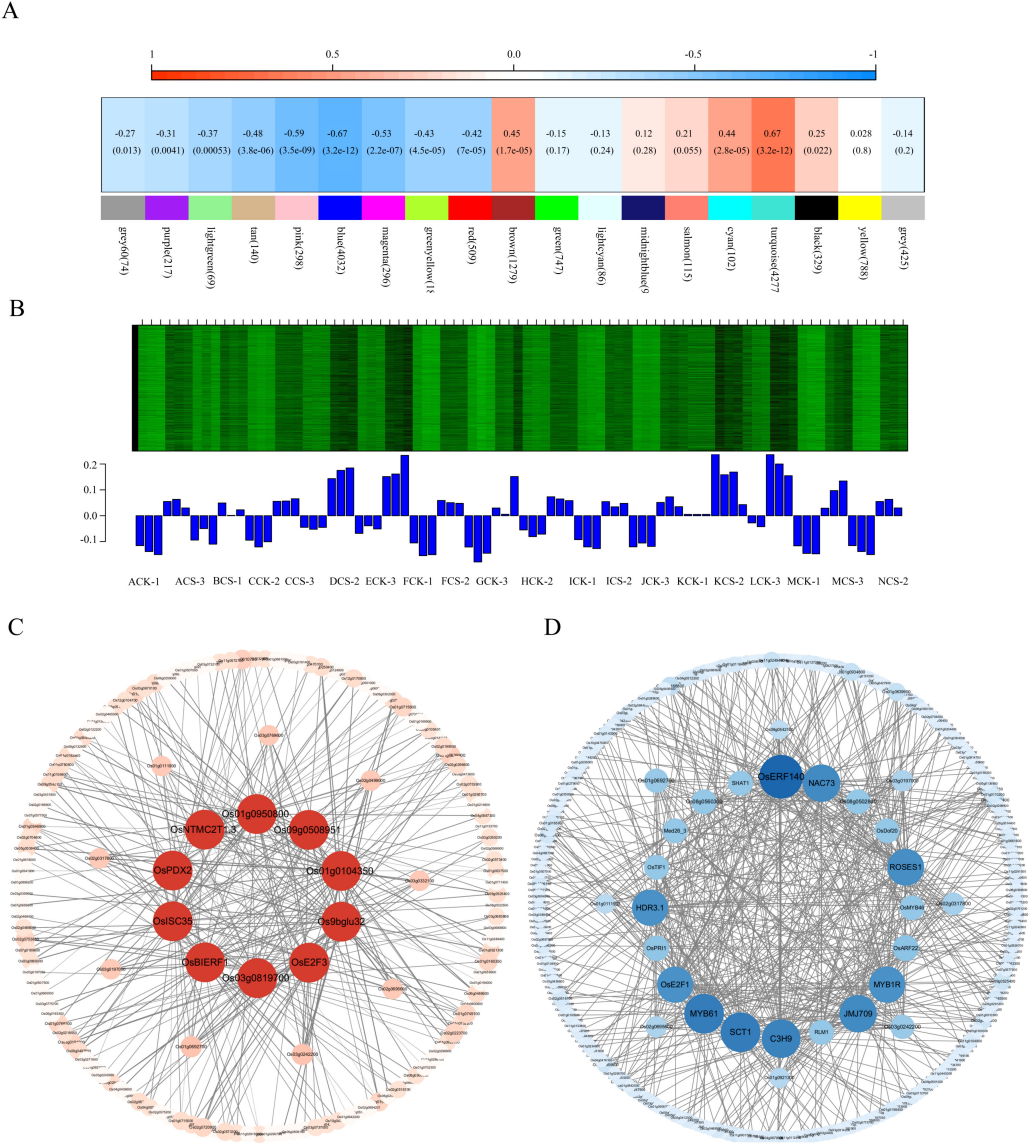
Co-expression network analysis and core regulatory network construction. **(A)** Module-trait correlation heatmap, the color depth indicates the correlation coefficient between the module and seed setting rate, **(B)** Expression patterns of module genes, **(C)** Interaction network of shared genes between candidate module and cold-tolerance gene set, nodes represent genes, and edges represent co-expression relationship, **(D)** Transcription factor-centered core regulatory network, nodes are transcription factors and edges represent co-expression relationship.

Given the established correlation between seed setting rate and cold tolerance in our preliminary findings, we performed module-trait association analysis through the MetWare Cloud Platform (https://www.metware.cn/) to identify cold-responsive modules. Notably, the blue module exhibited significant correlation with seed setting rate (**Figure 6A**). Subsequent expression profiling revealed marked upregulation of blue module genes under cold treatment, with significantly higher expression levels (*P*<0.05) in stress conditions compared to controls (**Figure 6B**). This cold-inducible expression pattern aligns with characteristic features of cold-responsive genes, suggesting the blue module’s critical role in cold stress adaptation mechanisms.

We performed GO enrichment analysis on the selected blue module (a candidate module associated with rice cold tolerance). In Molecular function, acetyltransferase activity, thioester hydrolase activity, and acylglycerol O-acyltransferase activity were significantly enriched. In Cellular component, microbody, peroxisome, and cytoplasmic microtubule showed significant enrichment. In Biological process, metabolic processes of cell wall macromolecules, phenylpropanoid metabolism, and cell wall biogenesis were significantly enriched (Additional file 10: **Supplementary Figure S4A**). Subsequent KEGG pathway enrichment analysis of the rice cold-tolerance candidate module revealed significant enrichment in:Valine, leucine and isoleucine degradation, Ubiquitin-mediated proteolysis, Plant hormone signal transduction, Phenylpropanoid biosynthesis, Peroxisome, Nucleotide metabolism, Cutin, suberine and wax biosynthesis, Circadian rhythm - plant, Biosynthesis of secondary metabolites, Biosynthesis of cofactors, Autophagy - other, Arachidonic acid metabolism (Additional file 10: **Figure 4B**). In summary, both the rice cold-tolerance candidate module and cold-resistant gene sets were significantly enriched in metabolic pathways closely related to cold tolerance, including cutin, suberine and wax biosynthesis, and plant hormone signal transduction. These results demonstrate that WGCNA can effectively construct biologically meaningful co-expression modules, and that this particular module may serve as a specific module for cold stress response in rice.

In the candidate modules, we calculated gene weights. The cold stress response module exhibited weighted values ranging from 0.1 to 0.34 (sorted in ascending order). Based on gene connectivity and overlap with a reference cold stress gene set, we selected 10 candidate cold stress-responsive genes: *OsISC35*, *OsBIERF1*, *OsE2F3*, *Os01g0950800*, *Os01g0104350*, *Os9bglu32*, *OsPDX2*, *Os09g0508951*, *OsNTMC2T1.3*, *Os03g0819700*. A corresponding gene expression regulatory network was constructed (**Figure 6C**), and functional annotations were provided for the candidate cold stress-responsive genes (Additional file 5: **Supplementary Table S5**). Among these genes: *OsISC35* encodes an FAD-linked sulfhydryl oxidase ALR. *OsBIERF1* is an ethylene-responsive factor. *OsE2F3* is involved in cell cycle regulation. *Os01g0950800* is an expressed protein. *Os01g0104350* belongs to a non-coding transcript. *Os9bglu32* is a β-glucosidase homolog, similar to the G. max hydroxynitrile lyase. *OsPDX2* is a pyridoxal biosynthesis protein. *Os09g0508951* is a hypothetical protein. *OsNTMC2T1.3* is an expressed protein. *Os03g0819700* is a conserved hypothetical protein. This translation ensures linguistic accuracy and academic rigor while maintaining clarity and precision in describing the methodology and functional annotations of the candidate genes.

### 3.6 Screening of transcription factors and construction of regulatory networks

Transcription factors play a key role in plant response to abiotic stress. In the candidate module of rice cold tolerance, a total of 288 transcription factors were identified, mainly from MYB, bHLH, bZIP, C2H2, NAC, AP2/ERF-ERF and WRKY7 families. Among them, there were 22 MYB family, 19 bHLH family, 14 bZIP family, 14 C2H2 family, 13 NAC family, 10 AP2/ERF-ERF family and 9 WRKY family. The gene expression network was visualized by Cytoscape software (**Figure 6D**). The first 20 transcription factors were selected as core genes to construct a regulatory network, and their functions were annotated (Additional file 6: **Supplementary Table S6**). The core genes include 4 MYB families, 4 zinc finger proteins, 2 AP2/ERF-ERF families, 1 B3-ARF family, 1 bHLH family, 1 CAMTA family, 1 E2F-DP family, 1 HB-BELL family, 1 IWS1 family, 1 Jumonji family, 1 NAC family, 1 DUF597 family and 1 SWIB/MDM2 domain protein.

Among them, *MYB1R* showed rhythmic expression under cold stress, which may regulate cold tolerance through circadian rhythm; *MYB61* is involved in cellulose synthesis and nitrogen utilization; *RLM1* and *OsMYB46* regulate secondary cell wall development; *OsERF140* and *SHAT1* belong to AP2/ERF family. *OsARF22* is an auxin response factor; *OsPRI1* regulates iron homeostasis; *OsDof20* and *OsTIF1* belong to the Dof and C2H2 families, respectively. *C3H9* is a zinc finger protein; *SCT1* is a Ca2^+^ sensing transcription factor; *OsE2F1* belongs to the E2F family; *ROSES1* regulates organ size; *med26–3* is a transcription elongation factor; *JMJ709* contains a JMJC domain; *NAC73* belongs to the NAC family; *HDR3.1* contains a LIM domain; *Os08g0560300* and *Os08g0502800* belong to PLATZ and SWI/SNF families, respectively.

## 4 Discussion

### 4.1 Correlation between seed setting rate at booting stage and cold tolerance

The booting stage is particularly vulnerable to cold stress, leading to severe yield losses due to impaired pollen development (Kuroki et al., 2007; Shi et al., 2025). In high-latitude regions like Heilongjiang Province, China, cold stress remains a major constraint for rice production. Our results confirmed that cold stress significantly reduced yield traits, with seed-setting rate showing the most pronounced decline (**Figure 1**), consistent with previous reports on pollen sensitivity to chilling (Saito et al., 2001; Hou et al., 2024; Shi et al., 2015). The higher relative seed-setting rate in cold-tolerant varieties reflects genotype-dependent cold resistance mechanisms during reproductive development.

### 4.2 Analysis of cold resistance mechanism in rice panicle formation

Transcriptome analysis revealed fundamental differences between cold-tolerant (6,240 DEGs) and cold-sensitive (7,996 DEGs) varieties. Cold-tolerant varieties activated photosynthesis (GO:0015979) and jasmonic acid response (GO:0009753), while cold-sensitive varieties upregulated phenylpropanoid metabolism (GO:0009698) and cell wall remodeling (**Figures 2E, F**), supporting the “stress tolerance-repair” model (Yamaguchi-Shinozaki and Shinozaki, 2006). From these DEGs

Accordingly, this study constructed a cold-tolerance gene set comprising 1,875 differentially DEGs, including genes specifically expressed in cold-tolerant varieties, and genes expressed in both cold-tolerant and cold-sensitive varieties but showing significantly higher differential expression levels in cold-tolerant varieties.The cold-tolerance gene set contained 14 known rice cold-tolerance genes: *OsDREB1B* (Dubouzet et al. 2003), *SNAC2* (Nakashima et al., 2007; Hu et al., 2008), *OsMYBS3* (Su et al., 2010), *OsTPP1* (Ge et al., 2008), *OsTPS8* (Li et al., 2011), *LIP19* (Shimizu et al., 2005), *OsbZIP38* (Liu et al., 2014), *OsMYB2* (Yang et al., 2012), *LTT7* (Liu et al., 2013), *OsTCP1* (Yang et al., 2013), *OsbZIP54* (Yang et al., 2021), *MAPKKK63* (Yeon-ju et al., 2019), *OsCAT1* (Zhu et al., 2022), *SAPK10* (Liu et al., 2021).

### 4.3 Regulatory roles of plant hormone signaling

KEGG enrichment analysis of the cold-tolerant gene set revealed significant enrichment in plant-pathogen interaction, biosynthesis of secondary metabolites, plant hormone signal transduction, MAPK signaling pathway, and starch and sucrose metabolism (**Figure 3B**). Among these, the plant hormone signal transduction pathway and MAPK signaling pathway were most closely associated with cold tolerance (Kim et al., 2024; Feng et al., 2024). In the gibberellin (GA) metabolic pathway, we identified eight differentially expressed bHLH family transcription factors (**Figure 4A**), including *OsbHLH1*, which has been functionally validated to confer cold tolerance (Wang et al., 2003). Within the abscisic acid (ABA) metabolic pathway, fourteen DEGs were detected (**Figure 4B**), with transcription factor *bZIP5* showing significant upregulation. Quantitative reverse transcription PCR analysis confirmed that *OsbZIP54* was strongly induced by cold stress and participates in plant abiotic stress responses (Yang et al., 2021).

### 4.4 Synergistic regulation of MAPK cascade and redox homeostasis

The MAPK signaling pathway contained 17 DEGs (**Figure 5**), among which *SAPK2* encodes a *SnRK2* family member belonging to the MAPK superfamily. Previous studies suggest that *MSK1* may be activated in response to stimuli that activate *SAPK2/p38* isoforms and the MAPK/ERK cascade, potentially contributing to cold tolerance pathways (Deak et al., 1998). Research demonstrated that *MKK1* (encoded by *OsCAT2* in this study) can promote rice seed germination under cold stress by coordinating MT- and ABI5-mediated signaling (Li et al., 2021). Notably, the activation of *MAPKKK17/18* and *CAT1* reveals a coupling mechanism between signal transduction and reactive oxygen species (ROS) scavenging. These findings are highly consistent with the previously proposed “ROS-calcium-MAPK” regulatory module (Mittler et al., 2022).

### 4.5 Modular regulatory mechanisms revealed by co-expression network analysis

WGCNA identified a seed-setting rate-associated blue module (**Figure 6A**). From this module, we selected the top 10 co-expressed genes, including *OsBIERF1* (mediating ethylene-ABA crosstalk) and *OsE2F3* (a cell cycle regulator; Zheng et al., 2022). The remaining genes (*OsISC35*, *Os01g0950800*, *Os01g0104350*, *Os9bglu32*, *OsPDX2*, *Os09g0508951*, *OsNTMC2T1.3* and *Os03g0819700*), though their functions remain uncharacterized, represent promising candidate genes potentially involved in cold-tolerance regulation that warrant further investigation.

Currently recognized transcription factor families associated with cold tolerance in rice include AP2/EREBP, MYB, WRKY, CBF, bZIP, ZEP, NAC, and bHLH (Agarwal et al., 2006). Our regulatory network analysis demonstrated that MYB (e.g., *MYB1R*) and NAC (e.g., *NAC73*) family members occupy central positions in the network (Additional file 6: **Supplementary Table S6**). Notably, *MYB1R* exhibits remarkable circadian expression patterns, suggesting its potential role in integrating circadian rhythms with low-temperature responses to achieve precise temporal regulation (Min et al., 2014). Among the 20 core transcription factors analyzed, one (*MYB1R*) has been functionally validated for cold tolerance, while the remaining factors (including *NAC73*, *OsBIERF1*, and *OsE2F3*) represent potential candidates whose roles in cold stress responses require further functional validation through experimental approaches.

## 5 Conclusion

This study systematically revealed the molecular regulatory mechanisms of rice cold tolerance through integrated analysis of yield components, transcriptomes, and co-expression networks. The seed-setting rate was confirmed as a reliable evaluation index, identifying Tengxi 144, Tengxi 180, and Yuanzi 2 as cold-tolerant varieties, with Daoguang, Xiannan 23, and Broom as cold-sensitive varieties. Transcriptome analysis detected 6,240 and 7,996 DEGs in cold-tolerant and cold-sensitive varieties respectively, from which 1,875 core cold-tolerance genes were identified. KEGG pathway analysis showed 44 DEGs participating in cold-tolerance-related pathways including ABA/GA metabolism and MAPK signaling, containing both known genes (*SAPK10*, *MAPKKK63*, *OsCAT1*, *OsbZIP54*) and 40 novel candidates. Co-expression network analysis of 14 japonica rice varieties generated 19 modules, with the seed-setting rate-associated blue module identified as a key cold-tolerance module. Final integration identified 10 candidate genes (including *OsISC35* and *OsBIERF1*) and 20 transcription factors (the known *MYB1R* plus 19 novel ones like *OsERF140* and *OsARF22*), providing crucial targets for understanding rice cold tolerance mechanisms and molecular breeding, while guiding future functional validation.

## Data Availability

The datasets presented in this study can be found in online repositories. The names of the repository/repositories and accession number(s) can be found in the article/**Supplementary Material**.
