# Supplementary material for: Integrated transcriptome and co-expression network analysis revealed the molecular mechanism of cold tolerance in japonica rice at booting stage
Source: Front Plant Sci. 2025 Jul 3;16:1629202. doi: 10.3389/fpls.2025.1629202 (PMC12268999; doi:10.3389/fpls.2025.1629202)
Supplement: Supplementary file 1 [file DataSheet1.zip › Additional file 1 Table S1.docx]

| Name | CK | CS | Relative seed-setting rate | Origin place | Subgroup |
| --- | --- | --- | --- | --- | --- |
| Tengxi 144 | 0.91 | 0.89 | 0.98 | Japan | P1 |
| Tengxi 180 | 0.91 | 0.81 | 0.89 | Japan | P1 |
| Yuanzi 2 | 0.91 | 0.74 | 0.81 | China | P3 |
| Jinxiandao 1 | 0.92 | 0.78 | 0.85 | China | MIX |
| Mudanjiang30 | 0.91 | 0.72 | 0.79 | China | P2 |
| Qiandaimian | 0.91 | 0.8 | 0.88 | Japan | P2 |
| Tiren | 0.95 | 0.75 | 0.79 | Japan | P1 |
| Liaojing 912 | 0.94 | 0.8 | 0.85 | China | MIX |
| Lige | 0.92 | 0.72 | 0.78 | Japan | P1 |
| Krista | 0.9 | 0.69 | 0.77 | Japan | P2 |
| Jinzao | 0.81 | 0.45 | 0.56 | China | P1 |
| Daoguang | 0.91 | 0.66 | 0.73 | Japan | P1 |
| Xiannan 23 | 0.83 | 0.41 | 0.49 | Korea | P2 |
| Broom | 0.91 | 0.37 | 0.41 | China | P3 |

Table S1 Specific information of the 14 japonica rice varieties
