# Supplementary material for: Integrated transcriptome and co-expression network analysis revealed the molecular mechanism of cold tolerance in japonica rice at booting stage
Source: Front Plant Sci. 2025 Jul 3;16:1629202. doi: 10.3389/fpls.2025.1629202 (PMC12268999; doi:10.3389/fpls.2025.1629202)
Supplement: Supplementary file 1 [file DataSheet1.zip › Additional file 2 Table S2.docx]

| Gene | Function annotation |
| --- | --- |
| *Os09g0460700* | gibberellin receptor, putative, expressed. |
| *Os05g0407550* | Hypothetical protein. |
| *Os09g0455500* | gibberellin receptor GID1L2, putative, expressed. |
| *Os06g0214300* | CXE carboxylesterase, putative, expressed. |
| *Os03g0790500* | gibberellin receptor GID1L2, putative, expressed. |
| *Os09g0461500* | gibberellin receptor GID1L2, putative, expressed. |
| *Os07g0162700* | gibberellin receptor GID1L2, putative, expressed. |
| *Os12g0136300* | Hypothetical protein. |
| *Os11g0705200* | GRAS family transcription factor domain containing protein, expressed. |
| *Os01g0842200* | ZOS1-16 - C2H2 zinc finger protein, expressed. |
| *Os11g0141500* | GRAS family transcription factor containing protein, expressed. |
| *Os06g0211500* | DELLA protein RGL3, putative, expressed. |
| *Os06g0610350* | GRAS family nuclear protein, Control of tillering, Regulation of panicle and. tiller development, Regulation of axillary meristem (AM) formation. |
| *Os06g0127800* | GAI-RGA-SCR GRAS) family protein, Brassinosteroid signaling. |
| *Os11g0124300* | Transcription factor, Asymmetric cell division involved in radial pattern. formation in roots, Control of stomatal development. |
| *Os06g0193400* | bHLH transcription factor, Tolerance to phosphate starvation. |
| *Os04g0493100* | Basic helix-loop-helix transcription factor, Defense response. |
| *Os01g0286100* | Phytochrome-interacting factor-like protein, Basic helix-loop-helix factor, Repression of seedling growth in the dark. |
| *Os10g0556200* | helix-loop-helix DNA-binding domain containing protein, expressed. |
| *Os02g0564700* | PTF1, putative, expressed. |
| *Os06g0164400* | Basic helix-loop-helix (bHLH) transcription factor, Negative regulation of seed dormancy, Preharvest sprouting (PHS) resistance. |
| *Os03g0759700* | bHelix-loop-helix transcription factor, putative, expressed |
| *Os05g0103000* | helix-loop-helix DNA-binding domain containing protein, expressed |
| *Os01g0286200* | Hypothetical protein |

Table S2 Differential genes enriched for gibberellin metabolic pathways
