# Supplementary material for: Integrated transcriptome and co-expression network analysis revealed the molecular mechanism of cold tolerance in japonica rice at booting stage
Source: Front Plant Sci. 2025 Jul 3;16:1629202. doi: 10.3389/fpls.2025.1629202 (PMC12268999; doi:10.3389/fpls.2025.1629202)
Supplement: Supplementary file 1 [file DataSheet1.zip › Additional file 3 Table S3.docx]

| Gene | Function annotation |
| --- | --- |
| *Os05g0213500* | Rice orthologue of the abscisic acid (ABA) receptor, Positive regulator of the ABA signal transduction pathway, Abiotic stress tolerance. |
| *Os05g0537400* | Clade A type 2C protein phosphatase, Negative regulation of ABA signaling,  Stomatal closure. |
| *Os03g0268600* | Similar to Protein phosphatase type 2C. |
| *Os01g0583100* | Similar to Protein phosphatase 2C. |
| *Os09g0325700* | Protein phosphatase 2C, biotic stress response, Early panicle development. |
| *Os02g0224100* | protein phosphatase 2C, putative, expressed. |
| *Os05g0457200* | Protein phosphatase 2C49, Negative regulation of salt stress response. |
| *Os04g0167875* | Protein phosphatase 2C-like domain containing protein. |
| *Os04g0167900* | Protein phosphatase 2C-like domain containing protein. |
| *Os07g0622000* | Serine/threonine protein kinase, Hyperosmotic stress response, Abscisic acid (ABA)-dependent gene regulation. |
| *Os05g0433100* | serine/threonine-protein kinase SAPK4, putative, expressed. |
| *Os03g0610900* | Serine/threonine protein kinase, Abscisic acid (ABA)-activated protein kinase,  Hyperosmotic stress response, ABA signal transduction. |
| *Os06g0719500* | bZIP transcription factor. |
| *Os12g0601800* | Similar to BZIP transcription factor family protein, expressed. |

Table S3 Differential genes enriched in the abscisic acid pathway
