# Supplementary material for: Integrated transcriptome and co-expression network analysis revealed the molecular mechanism of cold tolerance in japonica rice at booting stage
Source: Front Plant Sci. 2025 Jul 3;16:1629202. doi: 10.3389/fpls.2025.1629202 (PMC12268999; doi:10.3389/fpls.2025.1629202)
Supplement: Supplementary file 1 [file DataSheet1.zip › Additional file 5 Table S5.docx]

| Module | Gene | Function annotation |
| --- | --- | --- |
| blue | *OsBIERF1* | AP2-EREBP |
| blue | *OsE2F3* | E2F family transcription factor protein, putative, expressed. |
| blue | *Os01g0950800* | expressed protein |
| blue | *Os01g0104350* | Non-coding transcripts |
| blue | Os9bglu32 | Beta-glucosidase homologue, similar to G. max hydroxyisourat  e hydrolase, expressed |
| blue | *OsPDX2* | pyridoxal biosynthesis protein PDX, putative, expressed |
| blue | *Os09g0508951* | Hypothetical gene |
| blue | *OsNTMC2T1.3* | expressed protein |
| blue | *Os03g0819700* | expressed protein |
| blue | *OISC35* | FAD-linked sulfhydryl oxidase ALR, putative, expressed. |

Table S5 Candidate key genes in the candidate modules and their functional annotations
