# Supplementary material for: Integrated transcriptome and co-expression network analysis revealed the molecular mechanism of cold tolerance in japonica rice at booting stage
Source: Front Plant Sci. 2025 Jul 3;16:1629202. doi: 10.3389/fpls.2025.1629202 (PMC12268999; doi:10.3389/fpls.2025.1629202)
Supplement: Supplementary file 1 [file DataSheet1.zip › Additional file 6 Table S6.docx]

| Gene | granscripgion factor | Function annotation |
| --- | --- | --- |
| *OsERF140* | AP2/ERF-ERF | AP2 domain containing protein, expressed. |
| *OsARF22* | B3-ARF | auxin response factor-22. |
| *SCT1* | CAMTA | Ca ^2+^ -sensing transcription factor. |
| *MYB61* | MYB | regulate cellulose synthesis, promote nitrogen utilization. |
| *Os08g0560300* | PLATZ | zinc-binding protein, putative, expressed. |
| *RLM1* | MYB | Regulation of the development of secondary cell wall. |
| *OsMYB46* | MYB | Regulation of secondary wall biosynthesis. |
| *C3H9* | C3H | zinc finger C-x8-C-x5-C-x3-H type family protein, expressed. |
| *Med26_3* | IWS1 | transcription elongation factor protein, putative, expressed. |
| *ROSES1* | HB-BELL | Positive control of organ size. |
| *SHAT1* | AP2/ERF-ERF | Seed shattering through abscission zone (AZ) development. |
| *JMJ709* | Jumonji | jmjC domain-containing protein 4, putative, expressed. |
| *OsDof20* | C2C2-Dof | Zinc finger, Dof-type family protein. |
| *MYB1R* | MYB-related | MYB family transcription factor, putative, expressed. |
| *OsPRI1* | bHLH | Regulation of iron homeostasis. |

Table S6 Co-expression network with transcription factors as the core
