# Supplementary figures and images for: Integrated transcriptome and co-expression network analysis revealed the molecular mechanism of cold tolerance in japonica rice at booting stage
Source: Front Plant Sci. 2025 Jul 3;16:1629202. doi: 10.3389/fpls.2025.1629202 (PMC12268999; doi:10.3389/fpls.2025.1629202)

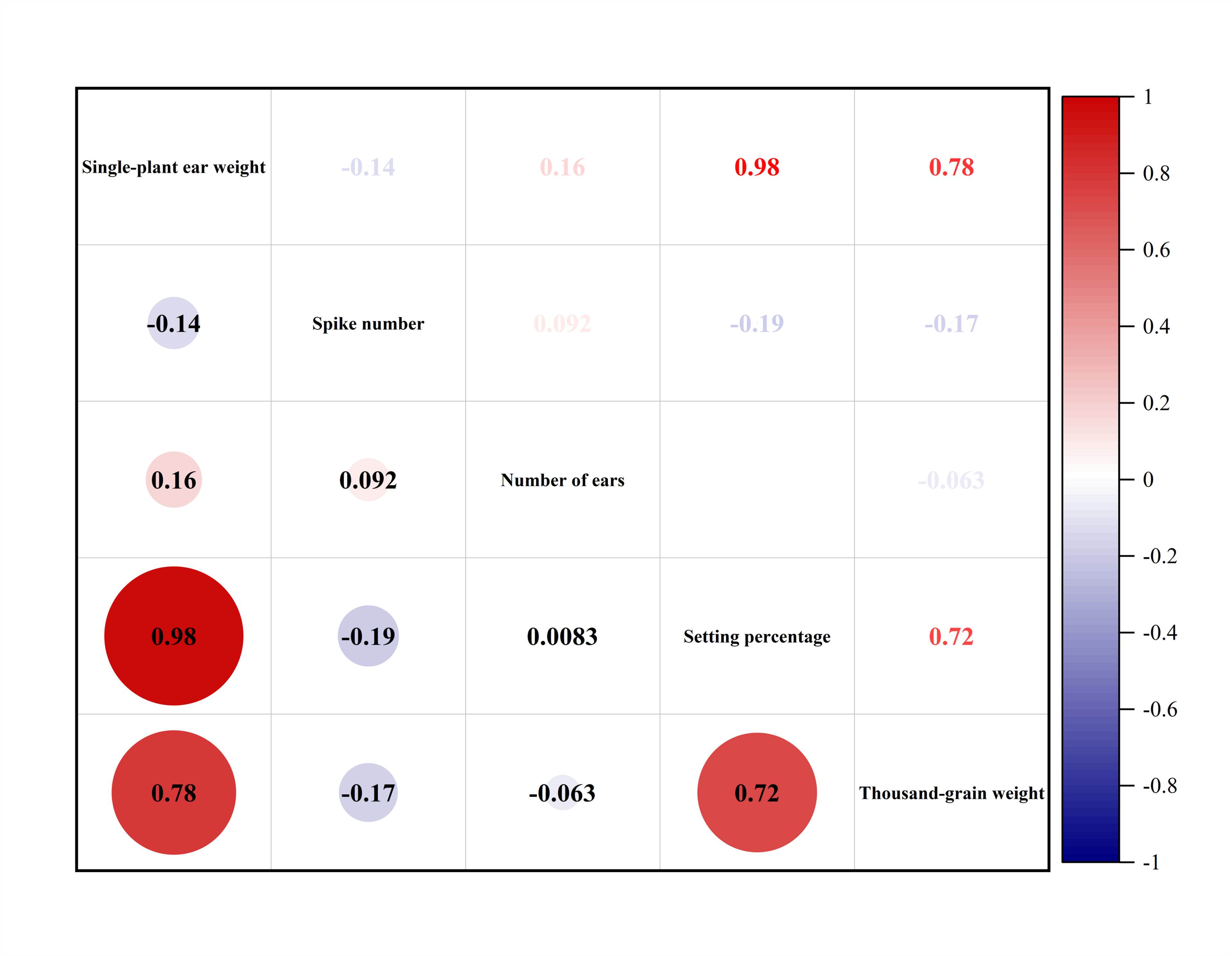

Supplement: Supplementary file 1 [file DataSheet1.zip › Additional file 7 Figure S1.tif]

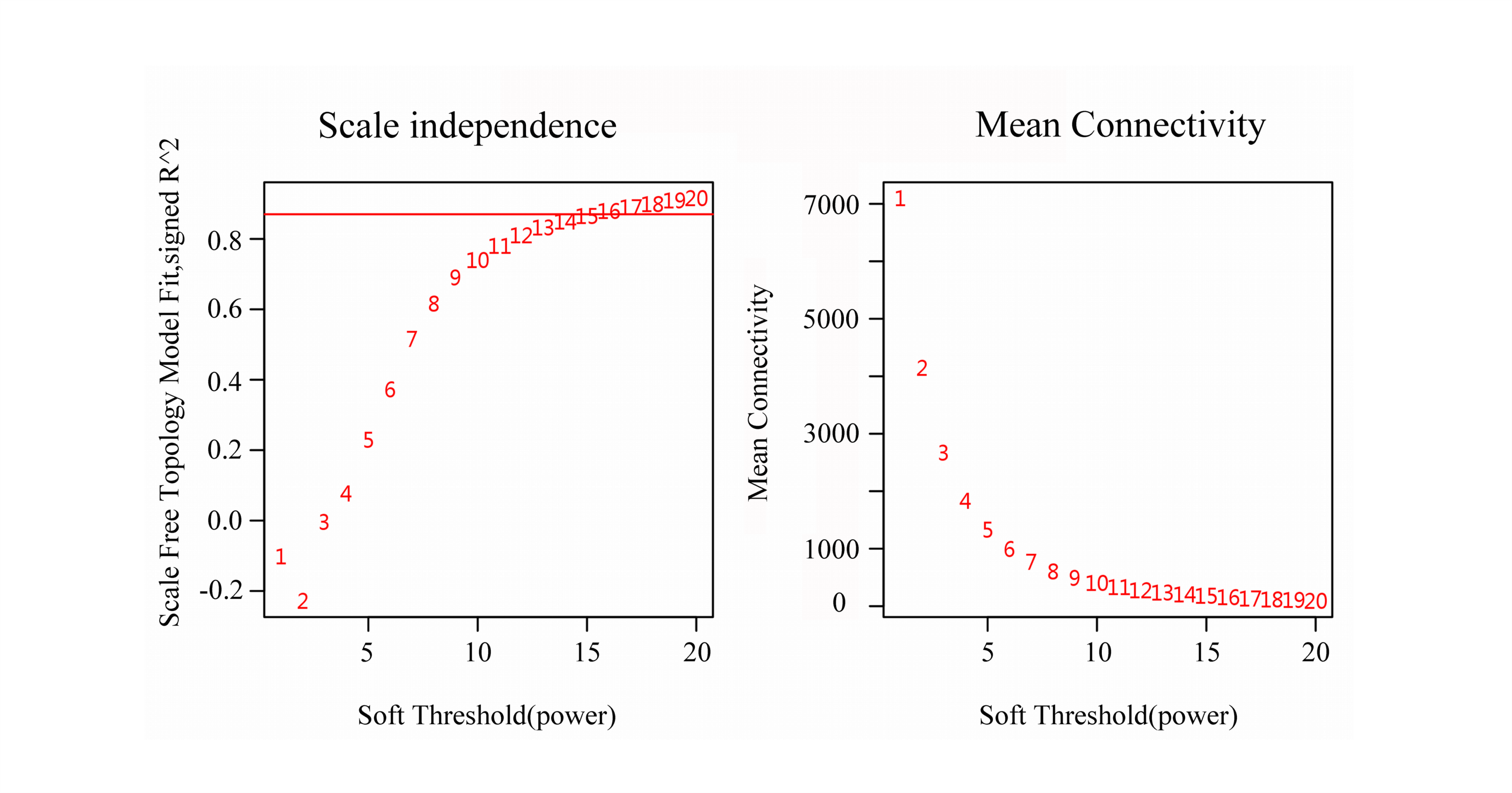

Supplement: Supplementary file 1 [file DataSheet1.zip › Additional file 8 Figure S2.tif]

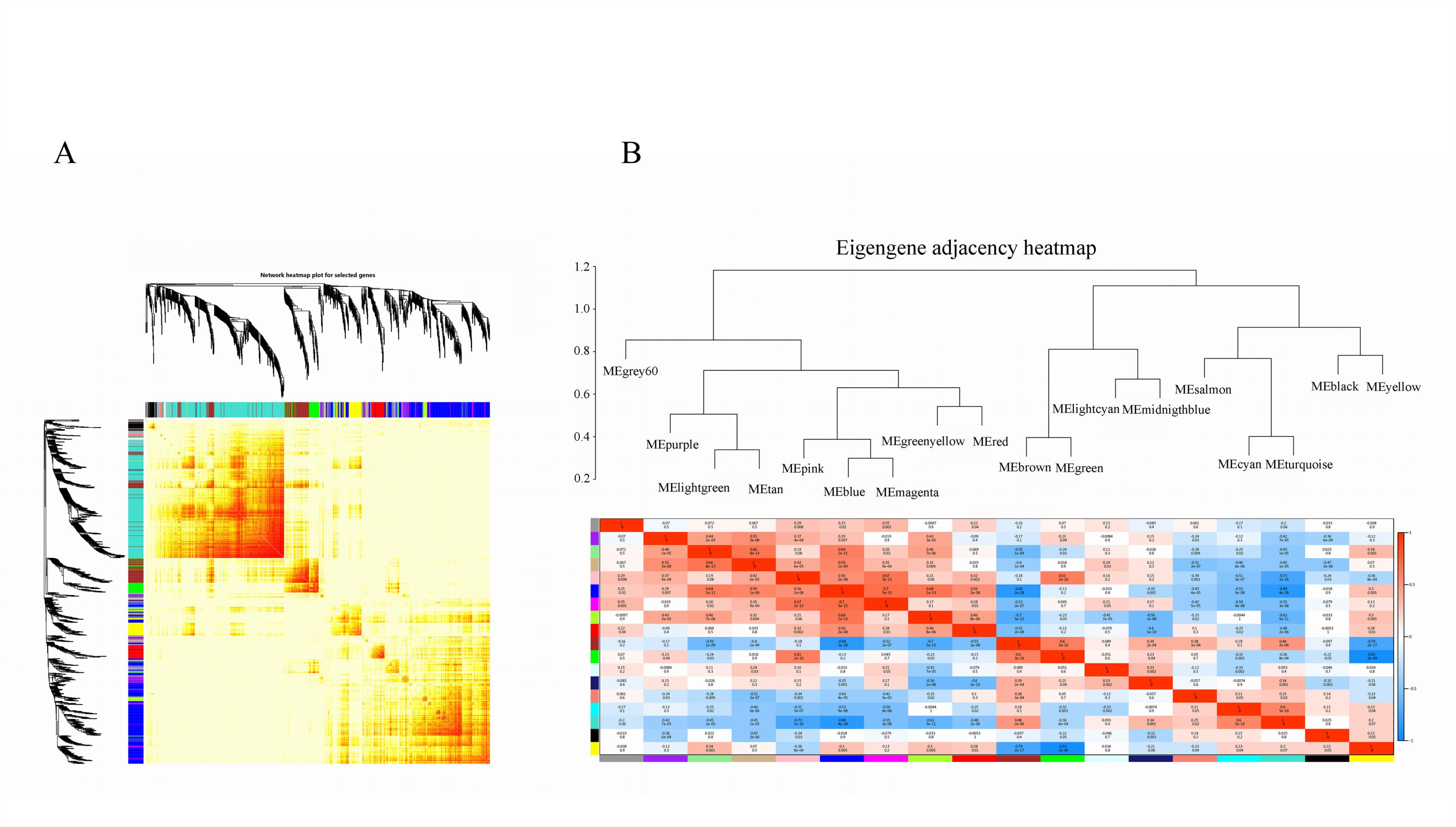

Supplement: Supplementary file 1 [file DataSheet1.zip › Additional file 9 Figure S3.tif]

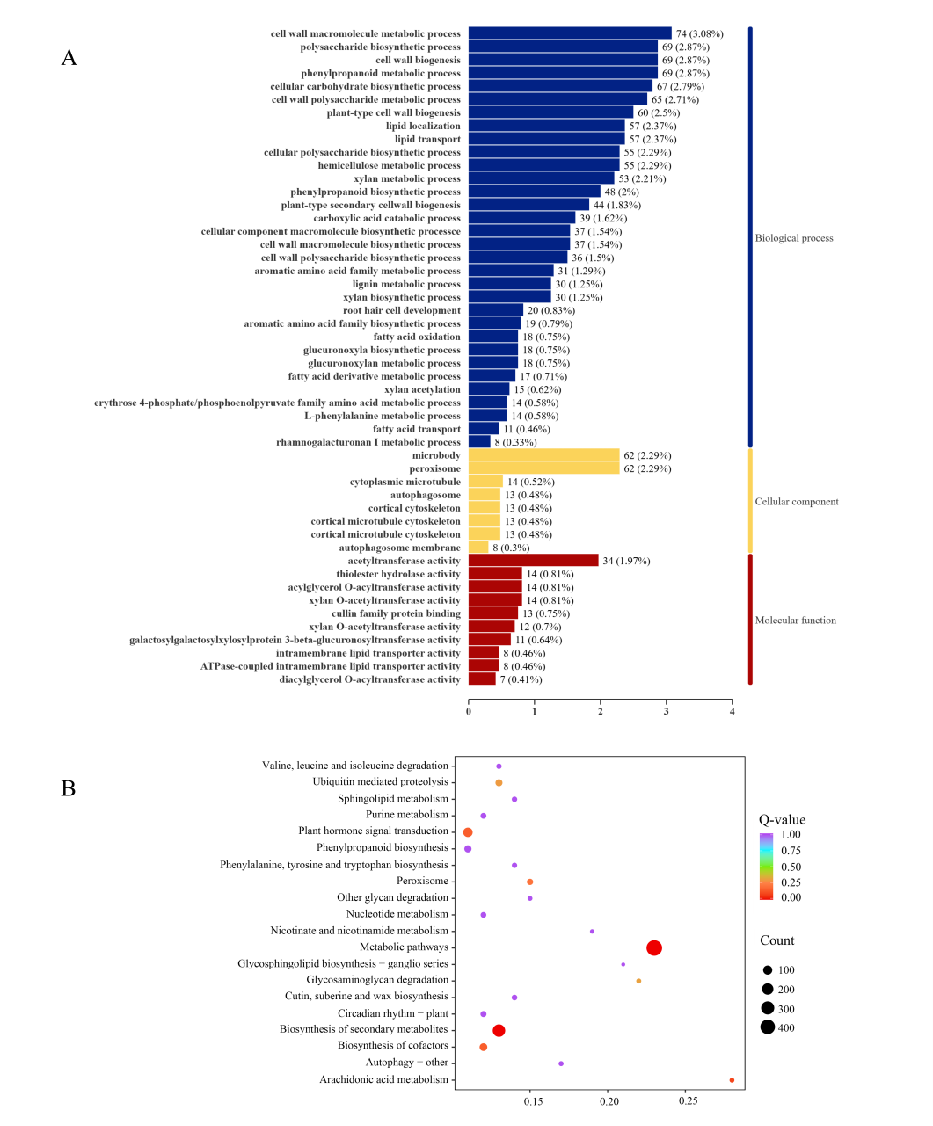

Supplement: Supplementary file 1 [file DataSheet1.zip › Additional file 10 Figure S4.tif]
